# Identifying genomic features by BLASTing through chromatin

**DOI:** 10.1186/1756-8935-6-S1-P43

**Published:** 2013-03-18

**Authors:** William KM Lai, Michael J Buck

**Affiliations:** 1 Department of Biochemistry and Center of Excellence in Bioinformatics and Life Sciences, SUNY Buffalo, 701 Ellicott St., Buffalo, NY 14203, USA

## Background

The sequencing of the human genome has revealed that the vast bulk of DNA sequence is devoted to regulatory regions. This regulatory information is spread throughout the genome and is responsible for guiding the complex cellular processes producing every human cell, tissue, and organ. The key to understanding this multifaceted network of genomic interactions has been the identification of common genomic features, ie. promoters and enhancers. However, finding these locations within the genome can be a laborious and expensive undertaking requiring site specific assays. Even more difficult is identifying entirely new classes of genomic features. To facilitate identification and characterization of new classes of genomic features we have developed Architecture Basic Local Alignment Search Tool (ArchBLAST).

## Results

The ArchBLAST algorithm uses the chromatin architecture at a user defined region or regions of interest and identifies all similar regions within the genome as defined by their shared chromatin architecture. ArchBLAST differs from other methods in that it uses both the amplitude and spatial arrangement of the chromatin modifications to score similarity. Importantly, ArchBLAST allows for the identification of subtypes of known genomic features and can accurately predict previously uncharacterized locations. ArchBLAST uses an innovative weighted profile generated from only the most informative chromatin datasets and then scores the entire genome. The accuracy of ArchBLAST has been validated with well characterized genomic features from yeast and humans. In addition, we demonstrate how ArchBLAST can identify novel transcription start sites in humans and show experimental evidence for their existence. We also show how ArchBLAST can uncover novel cell-type specific enhancers using only a few characterized enhancers as a template.

## Conclusions

We have shown that chromatin architecture is sufficient to identify and characterize genomic functional elements. ArchBLAST can incorporate any genomic dataset including transcription factor ChlP-seq and automatically selects the most informative datasets for the search. Additionally, ArchBLAST provides a flexible and expandable framework for identification of similar genomic elements using any user-defined criteria.

